# Hospital SARS-CoV-2 surveillance across inpatient testing, fever clinic, and general outpatient settings: positivity, Ct gradients, and short-lag warning signals

**DOI:** 10.3389/fpubh.2026.1908390

**Published:** 2026-09-30

**Authors:** Guanglin Cui, Ning Song, Wanhai Wang

**Affiliations:** 1 Clinical Laboratory, The First Affiliated Hospital of Zhengzhou University, Zhengzhou, Henan, China; 2 Key Laboratory of Laboratory Medicine of Henan Province, Zhengzhou, Henan, China; 3 Center for Reproductive Medicine, The First Affiliated Hospital of Zhengzhou University, Zhengzhou, Henan, China

**Keywords:** cycle threshold, early warning signal, hospital surveillance, inpatient testing, outpatient screening, SARS-CoV-2

## Abstract

**Background:**

Hospital-based SARS-CoV-2 surveillance needs to distinguish front-end detection settings from inpatient testing burden and evaluate whether outpatient virological signals can provide early warning for downstream hospital pressure.

**Methods:**

We conducted a single-center retrospective observational study using all SARS-CoV-2 nucleic acid test records from Zhengdong Campus, The First Affiliated Hospital of Zhengzhou University, between 20 December 2022 and 31 December 2025. The unit of analysis was individual test specimens, classified into fever clinic, general outpatient, and inpatient testing groups. Statistical analyses included chi-square tests, non-parametric tests, logistic regression, and time-lagged correlation analyses.

**Results:**

Among 24,751 tests, 5,000 were positive (overall positivity 20.2%). Positivity was highest in the fever clinic (26.9%), followed by general outpatient (23.5%) and inpatient settings (16.3%). Among 4,134 positive tests with available cycle threshold (Ct) values, median Ct was highest in inpatients (30.0) and lower in fever clinic (28.0) and general outpatient (27.0) settings. The proportion of general outpatient positives with Ct < 25 showed the strongest overall association with subsequent inpatient positivity at a lag of 2 biweekly intervals. Outpatient settings concentrated stronger virological signals, while inpatient testing carried the largest absolute burden of positive results.

**Conclusion:**

The general outpatient clinic functions as a meaningful front-end surveillance interface rather than a low-risk background category. Monitoring low-Ct enrichment in outpatient settings can provide a pragmatic early warning for short-term inpatient pressure, supporting broader outpatient-focused hospital infection prevention strategies.

## 1 Introduction

Hospital-based Severe Acute Respiratory Syndrome Coronavirus 2 (SARS-CoV-2) surveillance remains relevant to infection prevention and control because the operational questions are not limited to community incidence alone; hospitals also need to understand where infected patients are most likely to be detected, where diagnostic burden accumulates, and which service interfaces may provide the earliest practical signals for downstream pressure. Surveillance is a core component of infection prevention programs, and the growing use of electronic and laboratory data has expanded the possibilities for more timely hospital surveillance (1, 2). In China, these questions remain particularly relevant in the period after relaxation of the zero-COVID policy, when hospitals moved from tightly controlled screening pathways to a more dynamic post-policy phase (3–5).

Many hospital summaries of Coronavirus Disease 2019 (COVID-19) burden, however, focus primarily on inpatients or hospital-acquired cases (2, 6). This is important, but it can understate the role of outpatient-facing entry points, especially fever-clinic and non-fever outpatient pathways, in shaping hospital exposure risk and testing demand. In routine care, these pathways are not interchangeable: they differ in symptom profile, testing indication, and operational function, and therefore may also differ in positivity and virological signal. In the Chinese context, fever clinics have functioned as dedicated triage interfaces for potentially infectious respiratory illness, and their organization, standardization, and visit burden remain important operational issues in the post-pandemic period (7–9).

Cycle threshold (Ct) values are imperfect for individual-level decision-making and are influenced by assay, specimen quality, and testing context (10, 11). Nevertheless, at the population level, cross-sectional Ct distributions can reflect epidemic dynamics and may serve as early-warning indicators when interpreted cautiously and within a consistent analytic framework (10, 12–14). Recent studies also suggest that temporal trends in aggregated Ct values may help anticipate subsequent case burden, hospital admissions, or wave trajectories (15–17). For hospital surveillance, the potential value of Ct is less in assigning infectiousness to an individual patient than in identifying service settings in which lower-Ct positives become enriched before inpatient burden rises.

Using a large retrospective hospital testing dataset, we aimed to integrate three complementary perspectives: positivity across clinical settings, Ct-value gradients among positive tests, and time-lagged associations between outpatient Ct signals and inpatient detection burden. We focused specifically on whether the general outpatient clinic behaved merely as a background ambulatory category or as a meaningful front-end surveillance interface alongside the fever clinic.

## 2 Materials and methods

### 2.1 Study design and setting

This was a retrospective, record-based observational study conducted at Zhengdong Campus of The First Affiliated Hospital of Zhengzhou University, a large tertiary teaching hospital in Zhengzhou, Henan Province, China, with approximately 3,000 inpatient beds. The study used routine SARS-CoV-2 nucleic acid test records extracted from the hospital laboratory information system (LIS). Campus-wide patient attendance data are maintained separately in the hospital information system and were not included in the authorized study dataset. The study period was 20 December 2022 to 31 December 2025, spanning the local hospital period after relaxation of China’s COVID-19 control measures.

The unit of analysis was the test/specimen rather than the individual patient. Records were classified into three mutually exclusive clinical settings: fever clinic, general outpatient clinic, and inpatient. The inpatient category comprised patients from multiple departments who underwent SARS-CoV-2 testing during admission; it did not represent patients admitted because of COVID-19. The general outpatient category comprised non-fever ambulatory departments.

No formal *a priori* sample size calculation was performed. Because this was a retrospective record-based study, all eligible SARS-CoV-2 nucleic acid test records available in the LIS during the prespecified study period were included, thereby using the full set of available eligible records.

### 2.2 Laboratory procedures

Oropharyngeal or nasopharyngeal swab specimens were collected by trained nursing staff according to routine clinical procedures and placed immediately in commercially available viral transport medium. Viral transport media from more than one manufacturer were used during the study period as part of routine hospital procurement. Specimens were transported at room temperature to the clinical laboratory within 2 h of collection. Specimens tested on the day of collection were maintained at room temperature until processing, whereas specimens scheduled for testing the following day were stored at 2–8 °C until analysis.

Viral nucleic acid was extracted using a BG-Abot-96 automated nucleic acid extraction system (Shanghai BioGerm Medical Technology Co., Ltd., Shanghai, China). SARS-CoV-2 RNA was detected using the Novel Coronavirus (2019-nCoV) Nucleic Acid Detection Kit (PCR-Fluorescence Probing) (Shanghai BioGerm Medical Technology Co., Ltd., Shanghai, China) on an Applied Biosystems 7500 Real-Time PCR System (Applied Biosystems, Foster City, CA, United States). The assay targets the ORF1ab and N genes of SARS-CoV-2 and includes an internal control. Test validity and qualitative interpretation, including repeat testing of borderline amplification results when required, were determined according to the manufacturer’s instructions.

All testing was performed as part of routine clinical care by qualified clinical laboratory technologists following the laboratory’s standard operating procedures and the manufacturer’s instructions.

### 2.3 Data collection and management

Study data were collected retrospectively from routinely generated electronic laboratory records in the hospital LIS; no questionnaires, patient interviews, or study-specific case report forms were used. All eligible SARS-CoV-2 nucleic acid test records within the prespecified study period were electronically extracted from the LIS into a structured spreadsheet dataset. Extracted variables included testing date, ordering department, sex, age, qualitative SARS-CoV-2 test result, and ORF1ab and N-gene Ct values when available.

Clinical setting was assigned after data extraction using the ordering-department information, with records classified into the prespecified fever-clinic, general-outpatient, or inpatient categories. Direct patient identifiers, including names and patient identification numbers, were removed from the exported dataset before statistical analysis. Because the source data consisted of finalized, clinically verified laboratory reports, no study-specific questionnaires or additional manual chart abstraction were required, and missing values were not manually imputed or reconstructed.

### 2.4 Data definitions

The primary outcome for descriptive analyses was test positivity. The primary virological metric was the composite Ct value among positive records with available Ct data. For each positive specimen, Ct results were available for the ORF1ab and N gene targets; because these values were generally similar within the same specimen, their mean was used as the representative Ct value for analysis.

Three calendar periods were prespecified for setting-level comparisons: 2022–2023, 2024, and 2025. For the violin-plot comparison, Ct distributions were additionally viewed before and after 7 October 2023, a prespecified cut-off chosen to mark approximately 1 year after the first SARS-CoV-2-positive case identified at this hospital on 7 October 2022. Sex was categorized as male or female, and age was grouped as adults (≥18 years) and children/adolescents (<18 years).

### 2.5 Statistical analysis

Positivity was summarized as n/N (%) with Wilson 95% confidence intervals. Differences in positivity by clinical setting, sex, and age group were evaluated with chi-square tests. Normality of the composite Ct values was assessed using the Shapiro–Wilk test together with graphical inspection of the distributions. A two-sided *p* < 0.05 in the Shapiro–Wilk test was considered evidence of departure from normality. Because the Ct distributions showed significant departures from normality, Ct values were summarized using the median and interquartile range (IQR) and compared using non-parametric tests.

To estimate independent associations of clinical setting with test positivity, we fitted logistic regression models adjusting for calendar period, sex, and age group. Period-specific positivity models adjusted for sex and age group. Among positive records with available Ct data, additional logistic regression models examined the association between clinical setting and Ct < 25.

For temporal analyses, all records were aggregated into consecutive 14-day (biweekly) intervals. Biweekly test volumes and 4-biweek rolling positivity were used to visualize setting-specific dynamics. For early-warning analyses, four front-end exposure metrics were defined: fever-clinic median Ct, general-outpatient median Ct, fever-clinic Ct < 25 proportion among positives, and general-outpatient Ct < 25 proportion among positives. Two inpatient outcomes were evaluated: inpatient positivity and inpatient positive-record count.

Lagged associations were scanned across 0–4 biweekly lags using Spearman correlation. For heatmap interpretation, median-Ct associations were direction-reversed so that stronger early-warning signals always appeared as larger positive values. For quantitative support, standardized effects for inpatient positivity were estimated using robust ordinary least-squares models on z-scored series, and effects for inpatient positive counts were estimated using negative-binomial models with log(inpatient tests) as an offset. All analyses were interpreted as ecological surveillance analyses rather than patient-level transmission analyses.

Statistical analyses were performed using R software (version 4.5.3; R Foundation for Statistical Computing, Vienna, Austria). Logistic regression models were fitted to estimate adjusted odds ratios, Spearman correlation was used for lag analyses, robust ordinary least-squares models were used for standardized effect estimation, and negative-binomial regression models were used for count outcomes with log(inpatient tests) as an offset. Figures were generated in R. All tests were two-sided, and *p* < 0.05 was considered statistically significant.

## 3 Results

### 3.1 Study population and positivity across settings

A total of 24,751 SARS-CoV-2 test records were included, of which 5,000 were positive, yielding an overall positivity of 20.2%. Of all tests, 14,711 (59.4%) were from the inpatient testing population, 7,093 (28.7%) from the fever clinic, and 2,947 (11.9%) from the general outpatient clinic. Overall positivity differed significantly across settings (*p* < 0.001) and was highest in the fever clinic (26.9%, 1,910/7,093), followed by the general outpatient clinic (23.5%, 693/2,947) and inpatient testing (16.3%, 2,397/14,711) (Table 1). During 2022–2023, positivity was highest in inpatient testing (35.1%), whereas in 2024 and 2025 both outpatient settings exceeded inpatient testing. Despite this inversion, inpatient testing continued to contribute the largest share of all positive records in 2024 and 2025 because of substantially higher testing volume (Figure 1; Table 1).

**Table 1:** Positivity across inpatient, fever-clinic, and general-outpatient settings overall and by period.

Period	Patient category	Positive/total	Positivity, %	95% CI	Share of all tests, %	Share of all positives, %	*p* value
Overall	Inpatients	2397/14711	16.3	15.7–16.9	59.4	47.9	<0.001
Overall	Fever clinic	1910/7093	26.9	25.9–28.0	28.7	38.2	
Overall	General outpatient	693/2947	23.5	22.0–25.1	11.9	13.9	
2022–2023	Inpatients	1219/3476	35.1	33.5–36.7	36.1	39.0	<0.001
2022–2023	Fever clinic	1572/4982	31.6	30.3–32.9	51.7	50.3	
2022–2023	General outpatient	336/1170	28.7	26.2–31.4	12.2	10.7	
2024	Inpatients	707/4751	14.9	13.9–15.9	67.3	56.9	<0.001
2024	Fever clinic	236/1126	21.0	18.7–23.4	16.0	19.0	
2024	General outpatient	299/1180	25.3	22.9–27.9	16.7	24.1	
2025	Inpatients	471/6484	7.3	6.7–7.9	80.4	74.6	<0.001
2025	Fever clinic	102/985	10.4	8.6–12.4	12.2	16.2	
2025	General outpatient	58/597	9.7	7.6–12.4	7.4	9.2	

Wilson 95% confidence intervals are shown. “Share of all tests” and “share of all positives” were calculated within each period. *p* values are from chi-square tests comparing the three patient categories within each period.

**Figure 1:**
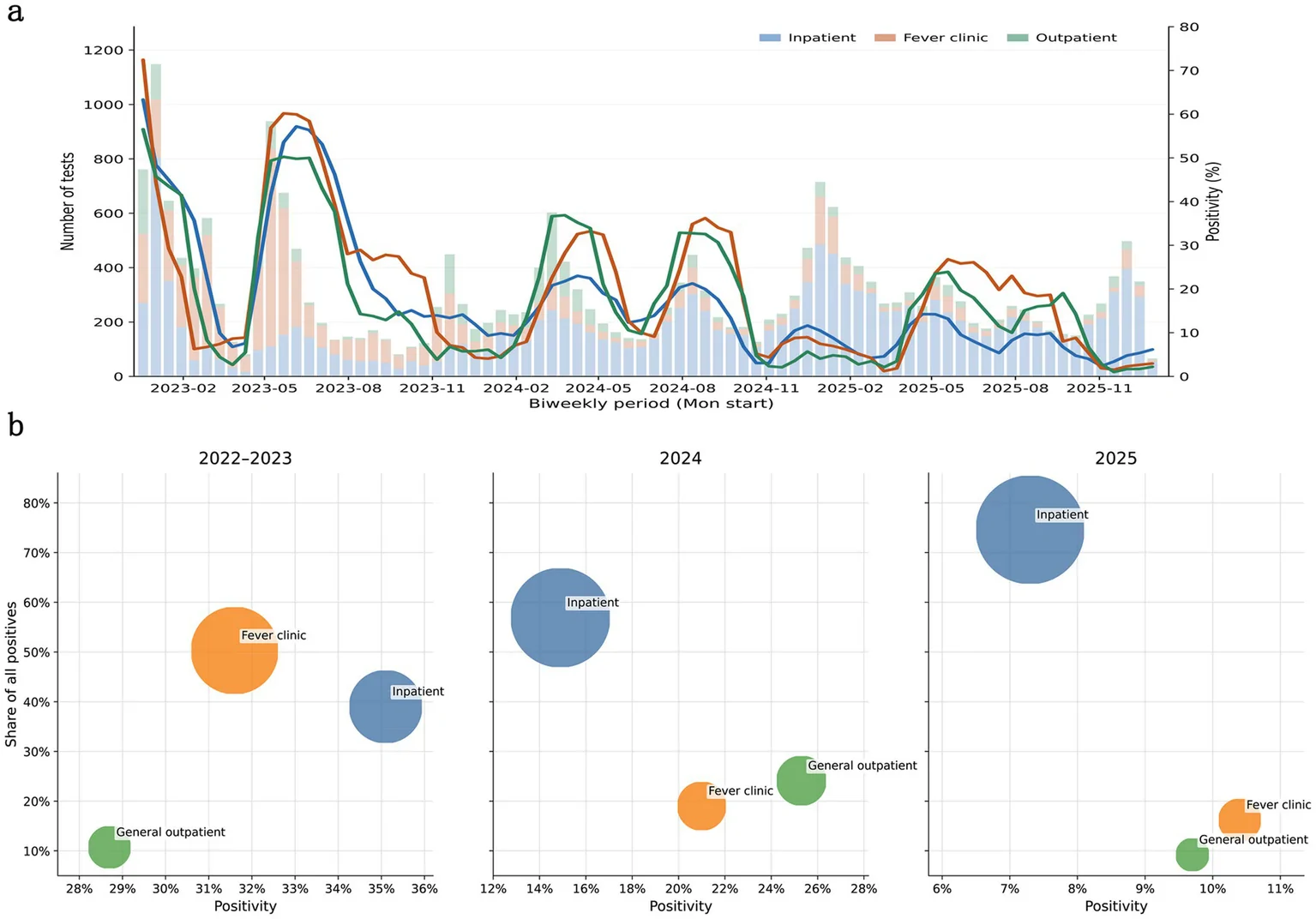
Temporal patterns of SARS-CoV-2 testing and positivity across patient categories. **(a)** Biweekly test volume and 4-biweek rolling positivity among inpatients, fever-clinic attendees, and general outpatients during the study period (20 December 2022 to 31 December 2025). Bars represent the number of tests in each biweekly period (left y-axis), and lines represent rolling positivity (right y-axis). Biweekly periods were defined using Monday start dates; the first period may be incomplete. **(b)** Period-specific bubble plots for 2022–2023, 2024, and 2025, showing setting-level positivity (x-axis), share of all positives (y-axis), and share of all tests (bubble area). These plots highlight differences between positivity, testing contribution, and absolute positive burden across care settings.

Positivity varied by sex and age group (Table 2). Overall, women had higher positivity than men (21.2% vs. 19.3%, *p* < 0.001), and the same pattern was observed in the fever clinic and general outpatient clinic, whereas inpatient testing showed slightly higher positivity in males (16.9% vs. 15.5%, *p* = 0.020). Age-group differences were modest overall, but adults had higher positivity than children/adolescents in inpatient testing (16.4% vs. 12.4%, *p* = 0.017) and general outpatient testing (24.6% vs. 15.2%, *p* < 0.001), while the difference was not significant in the fever clinic.

**Table 2:** Sex- and age-group-specific positivity within each patient population.

Domain	Population	Stratum	Positive/total	Positivity, %	95% CI	*p* value
Sex	Overall	Male	2546/13183	19.3	18.6–20.0	<0.001
Sex	Overall	Female	2454/11568	21.2	20.5–22.0	
Sex	Inpatients	Male	1399/8266	16.9	16.1–17.7	0.020
Sex	Inpatients	Female	998/6445	15.5	14.6–16.4	
Sex	Fever clinic	Male	876/3614	24.2	22.9–25.7	<0.001
Sex	Fever clinic	Female	1034/3479	29.7	28.2–31.3	
Sex	General outpatient	Male	271/1303	20.8	18.7–23.1	0.002
Sex	General outpatient	Female	422/1644	25.7	23.6–27.8	
Age group	Overall	Adults	4656/22992	20.3	19.7–20.8	0.504
Age group	Overall	Children/adolescents	344/1759	19.6	17.8–21.5	
Age group	Inpatients	Adults	2331/14180	16.4	15.8–17.1	0.017
Age group	Inpatients	Children/adolescents	66/531	12.4	9.9–15.5	
Age group	Fever clinic	Adults	1682/6194	27.2	26.1–28.3	0.274
Age group	Fever clinic	Children/adolescents	228/899	25.4	22.6–28.3	
Age group	General outpatient	Adults	643/2618	24.6	22.9–26.2	<0.001
Age group	General outpatient	Children/adolescents	50/329	15.2	11.7–19.5	

Adults were defined as age ≥18 years. *p* values are from within-population chi-square tests comparing sex strata or age-group strata.

### 3.2 Ct-value gradients across settings

Ct data were available for 4,134 of 5,000 positive records (82.7%). Ct completeness differed by setting, being 88.1% in the fever clinic, 80.7% in inpatient testing, and 74.6% in the general outpatient clinic (Table 3). Among positives with Ct values, median Ct was highest in inpatient testing at 30.0 (IQR 26.5–33.6) and lower in the fever clinic at 28.0 (23.0–31.5) and general outpatient clinic at 27.0 (23.5–31.0) (*p* < 0.001) (Figure 2b; Table 3).

**Table 3:** Ct-value distributions among positive records with available Ct data.

Period	Patient category	Ct available/positives	Ct completeness, %	Median Ct (IQR)	Ct <25, n/N (%)	95% CI	*p* value
Overall	Inpatients	1935/2397	80.7	30.0 (26.5–33.6)	305/1935 (15.8)	14.2–17.5	<0.001
Overall	Fever clinic	1682/1910	88.1	28.0 (23.0–31.5)	593/1682 (35.3)	33.0–37.6	
Overall	General outpatient	517/693	74.6	27.0 (23.5–31.0)	162/517 (31.3)	27.5–35.5	
2022–2023	Inpatients	802/1219	65.8	31.0 (27.2–34.0)	116/802 (14.5)	12.2–17.1	<0.001
2022–2023	Fever clinic	1352/1572	86.0	28.0 (22.5–31.5)	508/1352 (37.6)	35.0–40.2	
2022–2023	General outpatient	166/336	49.4	27.0 (22.0–31.1)	66/166 (39.8)	32.6–47.4	
2024	Inpatients	694/707	98.2	29.5 (25.5–33.9)	128/694 (18.4)	15.7–21.5	<0.001
2024	Fever clinic	234/236	99.2	27.5 (24.5–30.9)	64/234 (27.4)	22.0–33.4	
2024	General outpatient	295/299	98.7	27.5 (24.0–30.0)	81/295 (27.5)	22.7–32.8	
2025	Inpatients	439/471	93.2	30.0 (26.3–33.0)	61/439 (13.9)	11.0–17.4	0.005
2025	Fever clinic	96/102	94.1	28.6 (25.6–31.5)	21/96 (21.9)	14.8–31.1	
2025	General outpatient	56/58	96.6	28.8 (24.5–32.0)	15/56 (26.8)	17.0–39.6	

Ct analyses were restricted to positive records with an available composite average Ct value. *p* values are from Kruskal-Wallis tests comparing Ct distributions across the three patient categories within each period. Ct <25 was used as a pragmatic indicator of higher viral burden at the population level.

**Figure 2:**
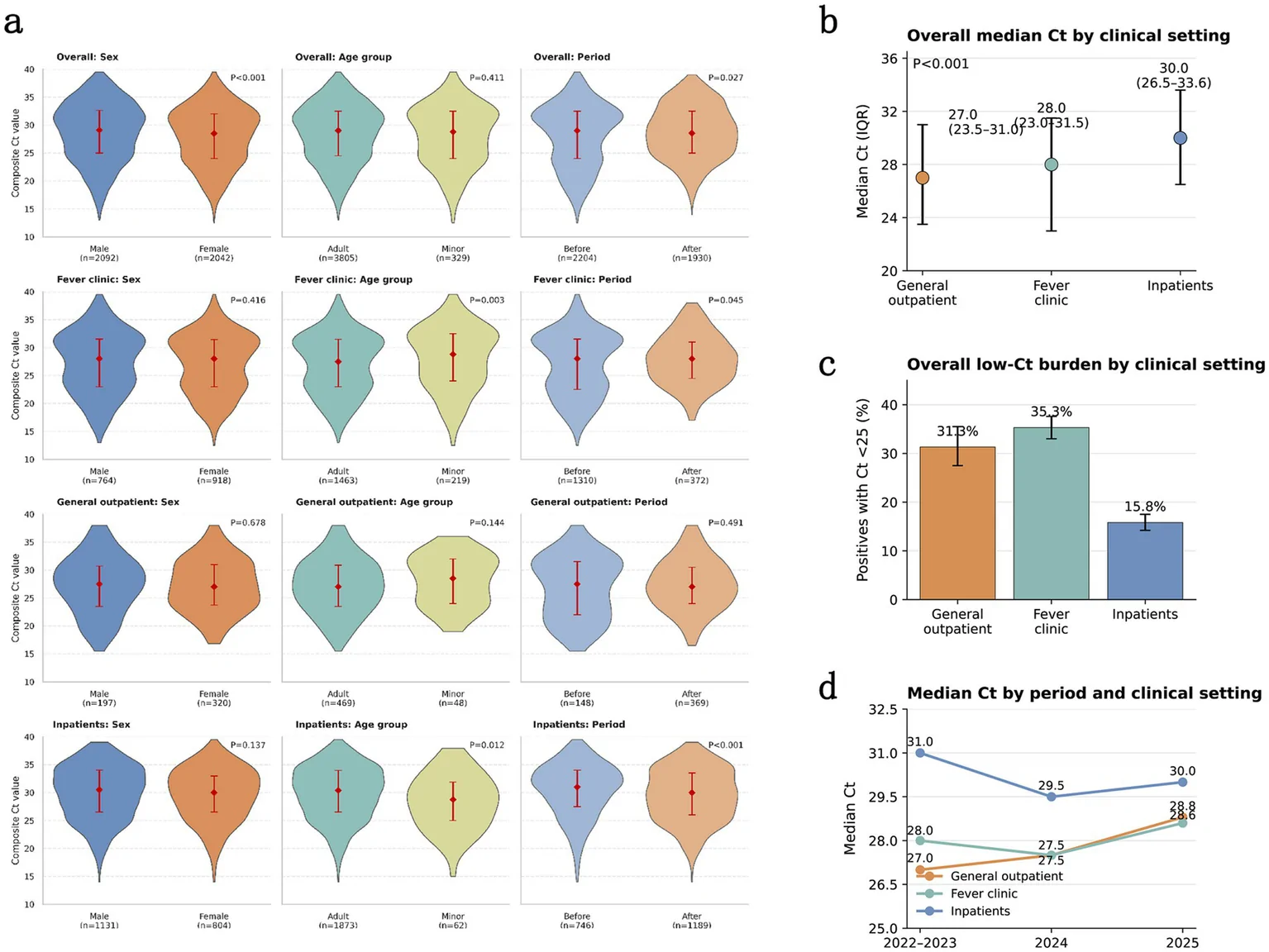
Ct-value distributions and summary gradients across clinical settings among SARS-CoV-2-positive records with available Ct values. **(a)** Violin plots showing composite Ct-value distributions stratified by sex, age group, and period in the overall cohort and within each clinical setting. Red dots and error bars indicate median and interquartile range (IQR). *p* values shown within each subplot represent between-group comparisons within that stratum. **(b)** Overall median composite Ct values by clinical setting, showing higher median Ct values in the inpatient testing population than in either outpatient setting (*p* < 0.001 for overall heterogeneity). Error bars indicate IQR. **(c)** Overall proportion of positive records with Ct < 25 by clinical setting, showing a lower low-Ct burden in the inpatient testing population than in outpatient settings. **(d)** Median composite Ct values by calendar period and clinical setting, showing persistently higher inpatient median Ct values across periods, with some attenuation of between-setting differences in 2024 and 2025. Composite Ct was defined as the mean of the ORF1ab and N gene Ct values for each positive specimen. The prespecified period split was 7 October 2023, approximately 1 year after the first SARS-CoV-2-positive case was identified in this hospital. The inpatient testing population represents hospitalized patients from multiple departments who underwent SARS-CoV-2 testing during admission, rather than patients hospitalized because of COVID-19.

The proportion of positives with Ct < 25 showed the same gradient: 15.8% in inpatient testing, 35.3% in the fever clinic, and 31.3% in the general outpatient clinic (Figure 2c; Table 3). This pattern persisted across calendar periods, although it was attenuated in 2024 and 2025. Within-stratum violin plots showed that setting-related Ct differences were more consistent than sex- or age-related differences (Figures 2a–d; Table 3).

### 3.3 Adjusted associations

In multivariable models adjusting for period, sex, and age group, both outpatient settings remained more likely than inpatient testing to yield a positive result overall, although the association was modest for the fever clinic (adjusted Odds Ratio (OR) 1.08, 95% Confidence Interval (CI) 1.00–1.17; *p* = 0.039) and stronger for the general outpatient clinic (adjusted OR 1.19, 95% CI 1.08–1.32; *p* < 0.001) (Table 4). Period-specific models showed an inversion over time: relative to inpatient testing, both outpatient settings were less likely to be positive in 2022–2023 but more likely to be positive in 2024 and 2025. Among positive records with available Ct values, both outpatient settings were more than twice as likely as inpatient testing to have Ct < 25 (fever clinic adjusted OR 2.64, 95% CI 2.23–3.13; general outpatient adjusted OR 2.41, 95% CI 1.92–3.03; both *p* < 0.001) (Table 4).

**Table 4:** Adjusted associations of patient category with test positivity and with Ct <25.

Outcome	Period	Comparison	Adjusted OR (95% CI)	*p* value
Test positivity	Overall	Fever clinic vs. inpatients	1.08 (1.00–1.17)	0.039
Test positivity	Overall	General outpatient vs. inpatients	1.19 (1.08–1.32)	<0.001
Test positivity	2022–2023	Fever clinic vs. inpatients	0.87 (0.80–0.96)	0.005
Test positivity	2022–2023	General outpatient vs. inpatients	0.76 (0.65–0.88)	<0.001
Test positivity	2024	Fever clinic vs. inpatients	1.53 (1.30–1.81)	<0.001
Test positivity	2024	General outpatient vs. inpatients	1.96 (1.67–2.28)	<0.001
Test positivity	2025	Fever clinic vs. inpatients	1.48 (1.18–1.85)	<0.001
Test positivity	2025	General outpatient vs. inpatients	1.37 (1.03–1.83)	0.031
Ct < 25 among positives	Overall	Fever clinic vs. inpatients	2.64 (2.23–3.13)	<0.001
Ct < 25 among positives	Overall	General outpatient vs. inpatients	2.41 (1.92–3.03)	<0.001

For overall positivity, the model adjusted for period, sex, and age group; for period-specific positivity, the model adjusted for sex and age group; for Ct <25, the model adjusted for period, sex, and age group among positive records with available Ct values. Inpatients were the reference category.

### 3.4 Short-lag associations between outpatient Ct signals and inpatient burden

Biweekly curves showed broadly synchronous waves across the three settings, so lag analysis focused on whether Ct-enriched outpatient signals carried additional short-term information for inpatient burden (Figure 3; Supplementary Table S1). In 2024, the strongest signal arose from the fever clinic, particularly fever-clinic Ct < 25 proportion at lag 0 biweeks for inpatient positivity (*r* = 0.655, *p* = 0.001) and inpatient positive-record count (*r* = 0.566, *p* = 0.005). In 2025, the strongest signal shifted to the general outpatient clinic, where Ct < 25 proportion showed the strongest associations with inpatient positivity at lag 2 biweeks (*r* = 0.563, *p* = 0.015) and inpatient positive-record count at lag 2 biweeks (*r* = 0.569, *p* = 0.014). In the overall best-lag summary, low-Ct burden performed more consistently than median Ct; the strongest overall associations were observed for general-outpatient Ct < 25 proportion with inpatient positivity at lag 2 biweeks (*r* = 0.307, *p* = 0.017) and inpatient positive-record count at lag 2 biweeks (*r* = 0.260, *p* = 0.045).

**Figure 3:**
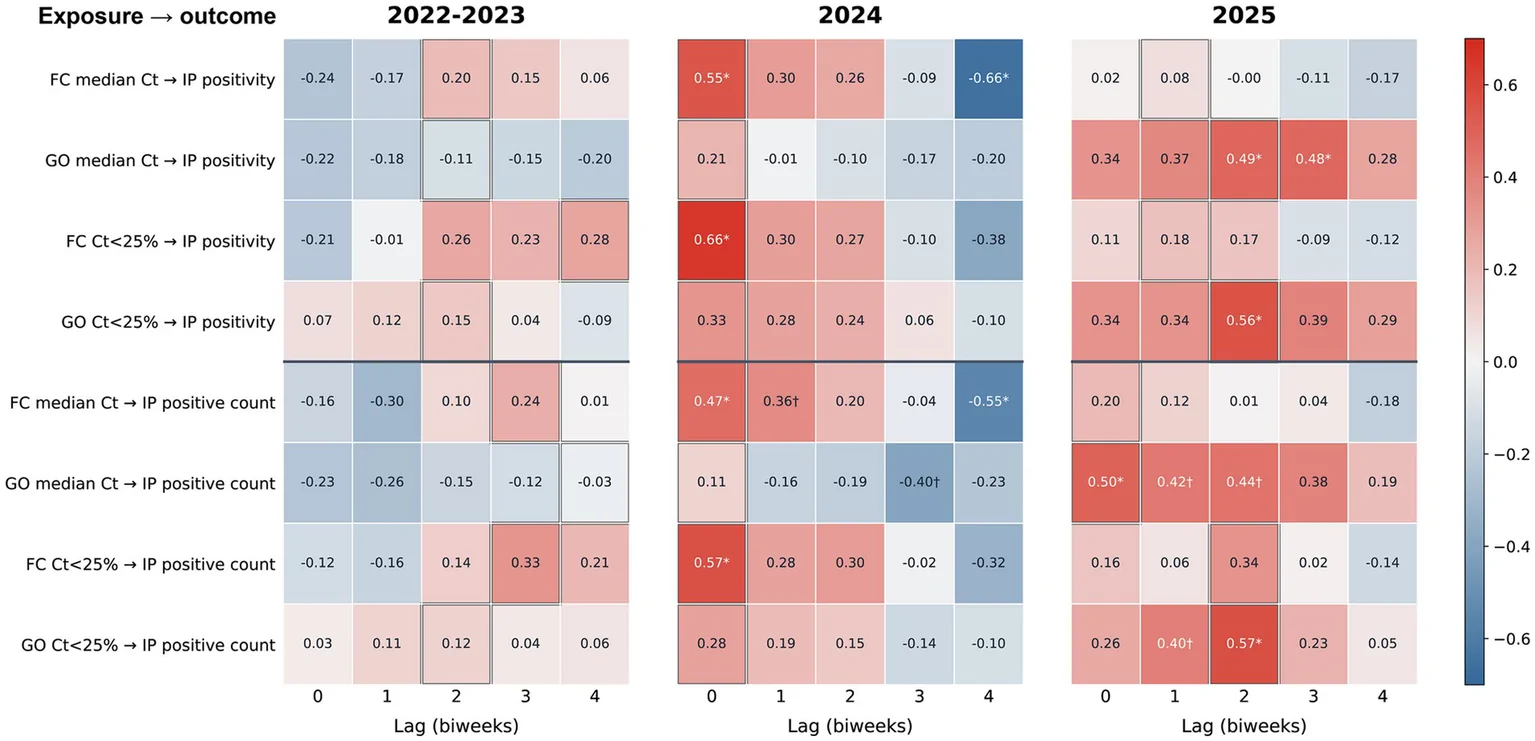
Period-stratified lag heatmap of front-end Ct signals and inpatient SARS-CoV-2 detection metrics. Heatmap cells show warning-oriented Spearman correlation coefficients for associations between lagged front-end Ct-based exposure metrics and inpatient outcomes across lags of 0–4 biweekly intervals, stratified by calendar period (2022–2023, 2024, and 2025). Front-end exposure metrics included fever-clinic (FC) median Ct, general-outpatient (GO) median Ct, FC Ct < 25%, and GO Ct < 25%. Inpatient outcomes included inpatient positivity (IP positivity) and inpatient positive count (IP positive count). For median Ct metrics, the sign of the correlation was direction-reversed so that warmer colors consistently indicate a stronger putative early-warning signal, that is, lower front-end Ct values associated with higher subsequent inpatient burden. The color scale represents the magnitude and direction of the warning-oriented Spearman correlation coefficient: deeper red indicates a stronger positive association, deeper blue indicates a stronger negative association, and colors near white indicate correlations close to zero. Columns labeled 0–4 indicate lags of 0 to 4 biweekly intervals. Black boxes indicate the best lag within each exposure–outcome pair. Asterisks denote *p* < 0.05, and daggers denote *p* < 0.10. Inpatients represent hospitalized patients from multiple departments who underwent SARS-CoV-2 nucleic acid testing during admission, rather than patients hospitalized because of COVID-19.

A trigger-style supplementary analysis supported the same pattern: mean inpatient positivity and inpatient positive-record counts 2 biweeks later were generally higher after intervals in which outpatient low-Ct burden reached moderate or high levels, particularly for signals arising from the general outpatient clinic (Supplementary Figure S1).

### 3.5 Outpatient composition

Among the two outpatient-facing settings, the fever clinic contributed 73.4% of all outpatient positive records overall and the general outpatient clinic 26.6%. In 2024, however, the balance reversed, with the general outpatient clinic contributing 55.9% of outpatient positives (Figure 4). A similar shift was observed for outpatient low-Ct positives: overall, the fever clinic contributed 78.5% and the general outpatient clinic 21.5%, whereas in 2024 the general outpatient clinic contributed 55.9% of outpatient low-Ct positives (Figure 4).

**Figure 4:**
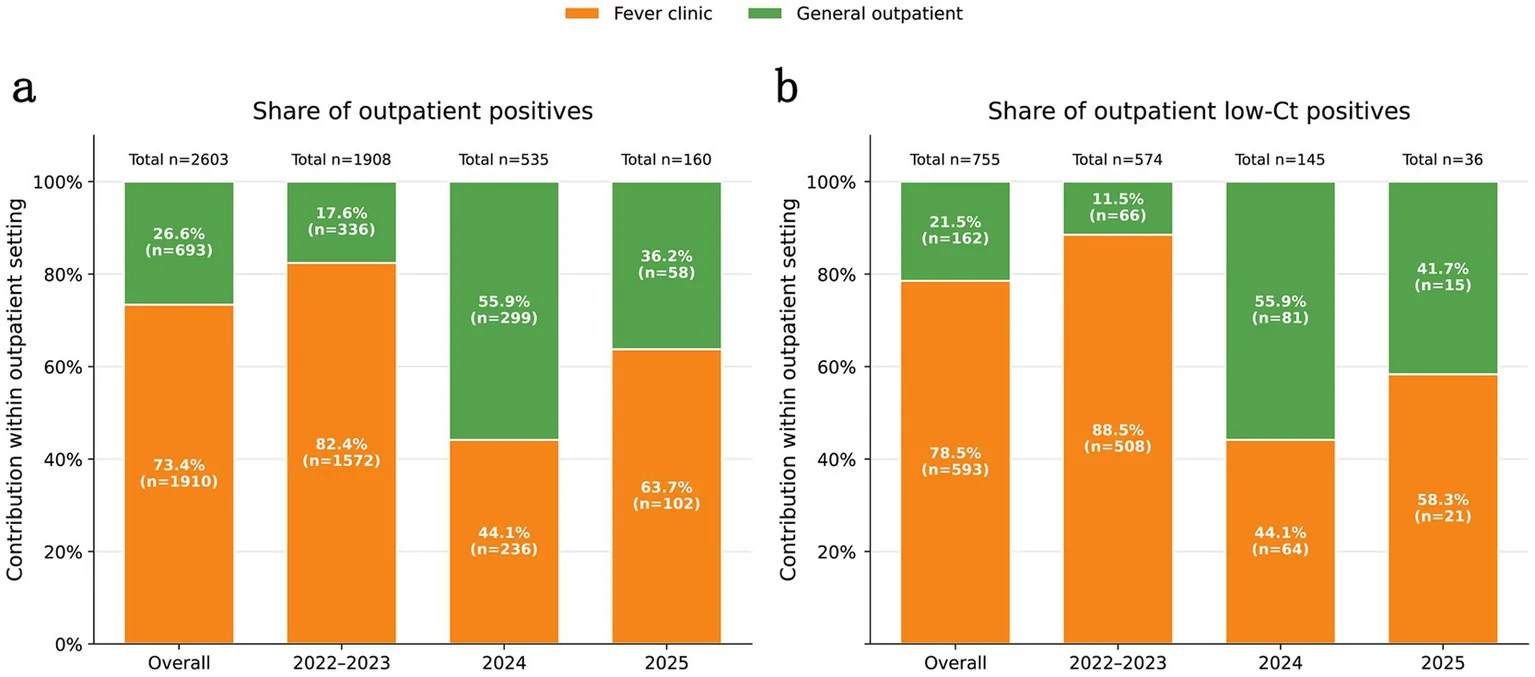
Composition of outpatient SARS-CoV-2 positives and outpatient low-Ct positives contributed by fever-clinic and general-outpatient settings overall and by period. Panel **(a)** shows the share of all outpatient positive records contributed by each outpatient setting. Panel **(b)** shows the share of outpatient low-Ct positives (Ct < 25). Bars are shown overall and for 2022–2023, 2024, and 2025; each bar segment is labeled with the within-period percentage contribution of fever-clinic and general-outpatient settings. The general outpatient clinic contributed an increasing share of both outpatient positives and outpatient low-Ct positives, particularly in 2024.

## 4 Discussion

This study highlights two complementary dimensions of hospital SARS-CoV-2 surveillance in a large Chinese tertiary hospital during the post-zero-COVID period. Outpatient-facing settings concentrated higher positivity and lower Ct values, whereas inpatient testing generated the greatest absolute burden of positive records because of sustained testing volume. These dimensions address different operational questions and therefore should not be conflated in hospital surveillance practice.

The temporal inversion from inpatient-dominant positivity in 2022–2023 to outpatient-dominant positivity in 2024–2025 suggests that surveillance assumptions should remain phase-sensitive after relaxation of China’s COVID-19 control measures. This shift aligns with broader epidemiological changes observed across China following the end of zero-COVID policy, during which detection rates of multiple respiratory pathogens increased substantially in both pediatric and hospital settings (18). Although fever clinics remain important triage interfaces in China (7–9), our data show that the general outpatient clinic should not be treated as a low-risk background category. Its positivity was substantial, its Ct profile resembled that of the fever clinic more than that of inpatient testing, and in 2024 it became the largest contributor to outpatient positives and outpatient low-Ct positives.

The Ct analyses add a second surveillance layer. Outpatient settings had a higher proportion of low-Ct positives than inpatient testing, and low-Ct burden performed better than median Ct in the lag analyses. The dominant signal was not fixed over time: fever-clinic low-Ct burden was more informative in 2024, whereas general-outpatient low-Ct burden was more informative in 2025. This phase dependence is plausible in a system where case mix, healthcare-seeking behavior, and testing indications evolve over time, and it supports cautious use of aggregated Ct metrics as pragmatic early-warning indicators rather than patient-level markers (15–17).

Sex- and age-specific differences in positivity were smaller and less consistent than the gradient across clinical settings. In our data, women had higher positivity than men in the fever clinic and general outpatient clinic, while inpatient testing showed a modest male excess; adults exhibited higher positivity than children/adolescents in both inpatient and general outpatient settings. These patterns align with evidence that sex and gender influence care-seeking behavior, testing indications, and case mix, resulting in elevated outpatient positivity among women (19–21). Age-related differences, including higher asymptomatic carriage among children than adults (22), have also evolved with variant circulation and progressive vaccination eligibility (23). In this study, such demographic variations are therefore best viewed as secondary descriptors rather than the primary surveillance signal.

In clinical practice, the general outpatient clinic should be included in routine SARS-CoV-2 surveillance rather than being viewed as a low risk background setting. During periods of increased SARS-CoV-2 activity, patients presenting to general outpatient departments with compatible symptoms may warrant timely testing and appropriate infection prevention measures according to local practice. The inpatient setting presents a different concern. Although positivity was lower among inpatients, this group accounted for the largest absolute number of positive results, indicating a substantial continuing burden for laboratory diagnosis and hospital infection prevention. Hospital surveillance may therefore benefit from considering both the concentration of positive specimens with Ct < 25 in outpatient settings and the absolute number of positive results among inpatients. The association between the outpatient proportion of specimens with Ct < 25 and inpatient detection over the following weeks further suggests that routinely generated laboratory data may help hospitals anticipate changes in testing demand and prepare for increases in inpatient burden. Because Ct values are influenced by assay and testing conditions, they should not be used alone to guide individual patient management (11). In hospital surveillance, however, aggregated Ct patterns may provide information that complements positivity and testing burden, particularly when changes are assessed over time (16, 17).

Several limitations should be acknowledged. First, the analysis was record-based rather than patient-based, so repeated testing may have influenced observed burdens. Second, the inpatient category comprised patients tested during admission rather than patients hospitalized because of COVID-19; accordingly, positivity in this group should not be interpreted as a direct measure of COVID-19 hospitalization risk or severity (24). Third, Ct completeness varied by setting and period, especially earlier in the study. Fourth, Ct values are assay- and context-dependent, and the lag analyses were ecological and cannot establish causal transmission pathways. Fifth, no formal *a priori* sample size calculation was performed because all eligible records during the study period were included. Although this maximized use of the available data, some subgroup and lagged analyses may still have had limited statistical power, particularly where the number of observations was small. As a single-center study, the generalizability of the findings to hospitals with different patient populations, testing practices, or service structures may also be limited. Nonetheless, the study also has notable strengths, including a large real-world dataset, continuous observation from late 2022 through 2025, and integration of positivity, Ct gradients, and short-lag analyses across clinically distinct hospital service interfaces in a post-policy Chinese setting. These findings align with recent observations from another Chinese tertiary hospital, which reported distinct age- and department-specific patterns of respiratory virus infections during the post-pandemic period (25). This consistency suggests that the shifts in SARS-CoV-2 detection across outpatient and inpatient settings may reflect broader post-policy changes in respiratory pathogen epidemiology within Chinese hospital systems.

## 5 Conclusion

Across 24,751 hospital SARS-CoV-2 tests, outpatient-facing settings yielded stronger virological signal whereas the inpatient testing population carried the greatest absolute burden. The general outpatient clinic behaved as a clinically meaningful front-end detection setting rather than a low-risk background category. These findings support broader outpatient-focused surveillance and suggest that monitoring low-Ct burden may help anticipate short-term inpatient pressure within hospital infection-prevention systems.

## Data Availability

The raw data supporting the conclusions of this article will be made available by the authors, without undue reservation.
