# Supplementary material for: Hospital SARS-CoV-2 surveillance across inpatient testing, fever clinic, and general outpatient settings: positivity, Ct gradients, and short-lag warning signals
Source: Front Public Health. 2026 Sep 30;14:1908390. doi: 10.3389/fpubh.2026.1908390 (PMC13661547; doi:10.3389/fpubh.2026.1908390)
Supplement: Supplementary file 1 [file Supplementary_file_1.DOCX]

**Supplementary Table S1. Best-lag summary for associations between front-end Ct signals and inpatient detection metrics.**

| **Outcome** | **Exposure setting** | **Exposure metric** | **Best lag (biweeks)** | **Spearman r** | **Spearman P** | **Effect type** | **Model effect** | **95% CI** | **Model P** | **N biweeks** | **Strongest period** | **Period consistency** |
| --- | --- | --- | --- | --- | --- | --- | --- | --- | --- | --- | --- | --- |
| Inpatient positivity | Fever clinic | Median Ct | 2 | 0.095 | 0.446 | Std. beta | 0.116 | -0.092 to 0.324 | 0.273 | 67 | 2024 | Consistent |
| Inpatient positivity | General outpatient | Median Ct | 0 | 0.180 | 0.168 | Std. beta | 0.094 | -0.101 to 0.288 | 0.346 | 60 | 2025 | Partially consistent |
| Inpatient positivity | Fever clinic | Ct <25 proportion | 2 | 0.273 | 0.025 | Std. beta | 0.174 | -0.056 to 0.403 | 0.138 | 67 | 2024 | Consistent |
| Inpatient positivity | General outpatient | Ct <25 proportion | 2 | 0.307 | 0.017 | Std. beta | 0.156 | -0.050 to 0.361 | 0.137 | 60 | 2025 | Consistent |
| Inpatient positive count | Fever clinic | Median Ct | 3 | 0.160 | 0.198 | IRR | 1.165 | 0.933 to 1.455 | 0.178 | 66 | 2024 | Consistent |
| Inpatient positive count | General outpatient | Median Ct | 0 | 0.060 | 0.651 | IRR | 1.087 | 0.920 to 1.285 | 0.325 | 60 | 2025 | Partially consistent |
| Inpatient positive count | Fever clinic | Ct <25 proportion | 2 | 0.182 | 0.141 | IRR | 1.206 | 0.964 to 1.509 | 0.101 | 67 | 2024 | Consistent |
| Inpatient positive count | General outpatient | Ct <25 proportion | 2 | 0.260 | 0.045 | IRR | 1.179 | 0.948 to 1.466 | 0.140 | 60 | 2025 | Consistent |

Notes: Heatmap correlations were warning-oriented: for median Ct metrics, lower Ct values were direction-reversed so larger positive values indicate stronger early-warning signals. Best lag was defined as the lag (0–4 biweeks) with the largest warning-oriented Spearman r in the overall series; ties were resolved in favour of the shorter lag. Positivity models use robust OLS on z-scored variables (reported as standardized beta). Count models use negative-binomial regression with log(inpatient tests) as offset (reported as IRR per 1-SD warning increase). A separate supplementary spreadsheet contains the complete lag-scan results across all periods and lags.

Figure S1

Supplementary Figure S1. Mean inpatient outcomes 2 biweeks after stratified front-end low-Ct burden in fever-clinic and general-outpatient settings. Points show mean inpatient positivity or positive-record count 2 biweeks later; error bars indicate 95% CI. Front-end low-Ct burden was grouped as none (0%), low (>0–<25%), moderate (25–<50%), or high (≥50%). Subsequent inpatient burden was generally higher after intervals with moderate or high front-end low-Ct burden.
